# Cybernic treatment with wearable cyborg Hybrid Assistive Limb (HAL) improves ambulatory function in patients with slowly progressive rare neuromuscular diseases: a multicentre, randomised, controlled crossover trial for efficacy and safety (NCY-3001)

**DOI:** 10.1186/s13023-021-01928-9

**Published:** 2021-07-07

**Authors:** Takashi Nakajima, Yoshiyuki Sankai, Shinjiro Takata, Yoko Kobayashi, Yoshihito Ando, Masanori Nakagawa, Toshio Saito, Kayoko Saito, Chiho Ishida, Akira Tamaoka, Takako Saotome, Tetsuo Ikai, Hisako Endo, Kazuhiro Ishii, Mitsuya Morita, Takashi Maeno, Kiyonobu Komai, Tetsuhiko Ikeda, Yuka Ishikawa, Shinichiro Maeshima, Masashi Aoki, Michiya Ito, Tatsuya Mima, Toshihiko Miura, Jun Matsuda, Yumiko Kawaguchi, Tomohiro Hayashi, Masahiro Shingu, Hiroaki Kawamoto

**Affiliations:** 1 Departments of Neurology and Rehabilitation Medicine, National Hospital Organization Niigata National Hospital, 3-52 Akasaka, Kashiwazaki, Niigata 945-8585 Japan; 2 Center for Cybernics Research, University of Tsukuba, Tsukuba, Japan; 3 Department of Orthopedics and Rehabilitation, National Hospital Organization Tokushima National Hospital, Yoshinogawa, Japan; 4 Department of Physical Rehabilitation, National Center Hospital, National Center of Neurology and Psychiatry, Kodaira, Japan; 5 Division of Neurology, Department of Internal Medicine, Jichi Medical University, Shimotsuke, Japan; 6 Department of Neurology, Kyoto Prefectural University of Medicine, Kyoto, Japan; 7 Division of Child Neurology, Department of Neurology, National Hospital Organization Osaka Toneyama Medical Center, Toyonaka, Japan; 8 Institute of Medical Genetics, Tokyo Women’s Medical University, Shinjuku, Japan; 9 Department of Neurology, National Hospital Organization Iou National Hospital, Kanazawa, Japan; 10 Department of Neurology, Faculty of Medicine, University of Tsukuba, Tsukuba, Japan; 11 Department of Rehabilitation, Tokyo Women’s Medical University, Shinjuku, Japan; 12 Department of Pediatric Neurology, National Hospital Organization Hokkaido Medical Center, Sapporo, Japan; 13 Department of Rehabilitation Medicine II, School of Medicine, Fujita Health University, Tsu, Japan; 14 Department of Neurology, Tohoku University Graduate School of Medicine, Sendai, Japan; 15 Health Administration and Policy, Tohoku University Graduate School of Medicine, Sendai, Japan; 16 Human Brain Research Center, Kyoto University Graduate School of Medicine, Kyoto, Japan; 17 Department of Rehabilitation, National Hospital Organization Hokkaido Medical Center, Sapporo, Japan; 18 Graduate School of Humanities and Social Sciences, Shizuoka University, Shizuoka, Japan; 19 ALS/MND Support Center Sakura, Nakano, Japan; 20 CYBERDYNE Inc., Tsukuba, Japan; 21 Faculty of Engineering, Information and Systems, University of Tsukuba, Tsukuba, Japan

**Keywords:** Neuromuscular disease, Hybrid Assistive Limb (HAL), Cybernics, Gait exercise

## Abstract

**Background:**

Rare neuromuscular diseases such as spinal muscular atrophy, spinal bulbar muscular atrophy, muscular dystrophy, Charcot-Marie-Tooth disease, distal myopathy, sporadic inclusion body myositis, congenital myopathy, and amyotrophic lateral sclerosis lead to incurable amyotrophy and consequent loss of ambulation. Thus far, no therapeutic approaches have been successful in recovering the ambulatory ability. Thus, the aim of this trial was to evaluate the efficacy and safety of cybernic treatment with a wearable cyborg Hybrid Assistive Limb (HAL, Lower Limb Type) in improving the ambulatory function in those patients.

**Results:**

We conducted an open-label, randomised, controlled crossover trial to test HAL at nine hospitals between March 6, 2013 and August 8, 2014. Eligible patients were older than 18 years and had a diagnosis of neuromuscular disease as specified above. They were unable to walk for 10 m independently and had neither respiratory failure nor rapid deterioration in gait. The primary endpoint was the distance passed during a two-minute walk test (2MWT). The secondary endpoints were walking speed, cadence, and step length during the 10-m walk test (10MWT), muscle strength by manual muscle testing (MMT), and a series of functional measures. Adverse events and failures/problems/errors with HAL were also evaluated. Thirty patients were randomly assigned to groups A or B, with each group of 15 receiving both treatments in a crossover design. The efficacy of a 40-min walking program performed nine times was compared between HAL plus a hoist and a hoist only. The final analysis included 13 and 11 patients in groups A and B, respectively. Cybernic treatment with HAL resulted in a 10.066% significantly improved distance in 2MWT (95% confidence interval, 0.667–19.464; *p* = 0.0369) compared with the hoist only treatment. Among the secondary endpoints, the total scores of MMT and cadence at 10MWT were the only ones that showed significant improvement. The only adverse effects were slight to mild myalgia, back pain, and contact skin troubles, which were easily remedied.

**Conclusions:**

HAL is a new treatment device for walking exercise, proven to be more effective than the conventional method in patients with incurable neuromuscular diseases.

*Trial registration*: JMACTR, JMA-IIA00156

## Background

To perform voluntary movements, humans need motor intention, which is transmitted via signals from the brain through the nervous system, and finally to effector muscles. In 1928, Sherrington used the term ‘motor unit’ for the complex of a lower motor neuron and the muscle fibres innervated by its axon [1]*.* For voluntary movements, the motor units are controlled by descending tracts of the central nervous system. There are a number of diseases that impair voluntary movements, and can occur at specific sites from the central nervous system to the motor unit. Previous studies, such as Ramón y Cajal [2], showed that in animal models, damaged neurons in the central nervous system (CNS) did not regenerate and synapses did not reconnect. Therefore, it has been thought that, in contrast to peripheral lesions, lesions of the CNS (including motor neurones) are permanent and cannot be regenerated even by rehabilitation. Hence, most rehabilitation programs for patients with motor impairment have not directly aimed to restore the function but rather only used the residual function to acquire compensatory activities [3].

Neuromuscular diseases are a broadly-defined group of disorders that involve injury to or dysfunction of the motor unit [4]. Especially in degenerative neuromuscular diseases, which are mainly caused by rare genetic variations, it has been considered that no therapeutic process is effective unless the underlying cause is removed at an early stage, owing to the dogma established by early on by studies such as that of Ramón y Cajal [2]. In these neuromuscular diseases, any treatment has been considered unlikely to be effective when advanced muscle atrophy and bone and joint deformities reach a progressed state. These progressive neuromuscular diseases are associated with a constant loss of the residual function and the difficulty in regaining compensatory activities. Moreover, excessive exercise conducted to improve the motor function has been known to lead to a further functional decline [5, 6]. Therapies that include muscle strength training and endurance training in neuromuscular diseases remain controversial [7–11]. Hence, exercise therapy to restore the motor function has been considered dangerous and is not a common method of recovering function in these patients. For these reasons, there is a notable lack of safe and effective exercise programs for patients with neuromuscular diseases.

Therefore, until recently, only curative treatments based on the aetiology of neuromuscular diseases have attracted attention. Nevertheless, it has been shown that aetiologically-based treatments do not provide sufficient functional improvement in progressive neuromuscular diseases. For example, nusinersen—an antisense oligonucleotide—has been clinically tested for the treatment of spinal muscular atrophy (SMA), which is caused by a deficiency in the survival motor neuron (SMN) protein; the results showed that the effect of the treatment diminished as the disease progressed [12]. Furthermore, although leuprorelin—which inhibits the nuclear transportation of testosterone and the abnormal androgen receptor—has been clinically demonstrated to improve dysphagia in patients with bulbar spinal muscular atrophy (SBMA), there was no improvement in the ambulatory function [13]. While muscle biopsies in patients undergoing exon skip therapy to target the dystrophin gene with eteplirsen demonstrated the desired effect on protein synthesis in patients with the fatal neuromuscular disorder Duchenne muscular dystrophy (DMD), no sufficient relevant motor improvement was observed [14, 15]. Thus, even if drug developments are aetiologically successful, it is considered highly unlikely that lower motor neurons will re-innervate the muscle fibres and that the central nervous system will also regain control over the motor unit, including lower motor neuron. Therefore, the development of therapeutics that promote neuromuscular reconnection or remodulation has become an important research goal.

A member of our team, Dr Yoshiyuki Sankai, pioneered the innovative academic field known as ‘cybernics’, or the fusion of humans, robots, and information systems. Dr Sankai invented the wearable cyborg Hybrid Assistive Limb (HAL) based on the principle of cybernics [16–20]*.* Cybernics technology links the human nervous system and a robot through bioelectric signals such as motor unit potentials, which results in a dynamic state in which the wearer and HAL are functionally and physically connected. HAL functions in accordance with the motor intention of the wearer and internal ideal movement patterns as if it were a part of the body. The process that realizes the fusion of a human and HAL via the mutual exchange of neural information (e.g., motor unit potentials and proprioception) and dynamic mechanical information (e.g., coordinates, velocity, and angular velocity in limb segments) between the wearer’s nervous system and HAL was named “interactive biofeedback (iBF)” by Dr Sankai. Since its inception, HAL has been implemented into the society (e.g., HAL lumbar type for labour support in the workplace) as well as the medical field as the world's first cyborg-type robot [21].

Despite visual similarities, HAL is completely different from other exoskeletal robots [22, 23], which are not cybernics based. These non-cybernic exoskeletal robots repeat mechanical patterns or are under the physical control of the wearer. In the case of HAL, the wearer performs voluntary movements, in which the neuromuscular and central nervous systems work to produce the intended movement of the integrated HAL and the wearer’s body. HAL is synchronized with the intention to move in accordance with the motor unit potentials produced by a command from the CNS. In this way, the iBF loop is created between the wearer and HAL [18, 24–30] as follows: [brain ≫ spinal cord ≫ motor nerves ≫ (HAL + musculoskeletal system)] and [HAL ≫ musculoskeletal system ≫ sensory nerves ≫ spinal cord ≫ brain]. Using HAL, this loop can be easily repeated without increasing the neuromuscular system’s excessive load and fatigue. The physician or operator can intervene in this transmission loop by tuning the parameters embedded in HAL*.* Furthermore, it is believed that the return of sensory signals from the proprioceptors in the muscles, joints, and skin to the CNS is of great importance for neuroregeneration [31, 32].

In the present study, we propose that cybernics (i.e., the iBF created by the union of HAL and the nervous and musculoskeletal systems of the wearer) enables the regeneration of the impaired motor function. This neuroplasticity [33] would result in the reconnection of the CNS to the motor units [34] and the reconnection of motor neurons to muscle fibres. Thus, we hypothesized that the strengthening and adjusting of the synaptic connections in the neuromuscular and central nervous systems can be achieved via the iBF loop and may therefore result in neuroregeneration and regaining of the motor function, i.e., motor learning. At the beginning, we planned two clinical trials (RCTs) to assess the efficacy of iBF in neuroregeneration [30, 35, 36]: (1) NCY-2001, an RCT conducted to confirm whether iBF can induce neuroplasticity in case of the lesions of the CNS (i.e., lesions of the brain and spinal cord, located above the level of the motor units; spastic paraplegia), and (2) NCY-3001, an RCT conducted to confirm whether iBF is effective when motor units are damaged. When motor units are damaged, HAL needs to effectively detect weak and sparse motor unit potentials and compensate for very weak muscle power. Considering that HAL for medical use (lower limb type), which was originally developed for neuromuscular diseases, could also be helpful in a wider range of diseases and levels of progression (e.g., acute spinal cord injury and cerebrovascular disease to chronic disease), and given the lack of therapeutics that promote neuroregeneration, we sought to test the efficacy of HAL (NCY-3001) first in cases of slowly progressive rare neuromuscular diseases in which motor units were damaged (e.g., SMA, SBMA, muscular dystrophy, Charcot-Marie Tooth disease [CMT], distal myopathy, sporadic inclusion body myositis, congenital myopathy, and amyotrophic lateral sclerosis [ALS]).

## Methods

### Study design and patients

The NCY-3001 clinical trial constitutes an open-label, multicentre, randomised, controlled trial, using a crossover design to evaluate the safety and efficacy of an intermittent cybernic treatment with HAL to improve the gait function in patients with neuromuscular diseases. A crossover design was used to reduce the required sample size, which was an important issue because the target diseases are very rare. A requirement for the crossover design is that the symptoms of the disease (e.g., gait impairment) are stable over the time period of the study. To facilitate the recruitment of patients, the trial was conducted at nine clinical sites in Japan between March 6, 2013, and August 8, 2014. The general eligibility criteria were as follows: age ≥ 18 years; diagnosis confirmed based on diagnostic criteria for SMA, SBMA, ALS, muscular dystrophy, CMT, distal myopathy, sporadic inclusion body myositis, or congenital myopathy; no rapid changes in gait symptoms for three months prior to the start of the trial; an inability to walk 10 m independently without the use of an assistive device such as a cane or a walker. Eligible patients were further screened with the following exclusion criteria: exercise limitations owing to cardiac and/or respiratory failure; skeletal deformities such as osteoarthritis of the hip, knee, or spine, and scoliosis; severe osteoporosis; severe tendency to bleed; reliance on a ventilator; serious hepatic or renal disorder; incurable neoplasm. The following criteria specific to the use of HAL were also evaluated: body weight 40–100 kg; height 150–190 cm; healthy skin for the placement of the electrodes; ability to move the hip and knee joints using the cybernic voluntary control (CVC) mode of HAL; generation of a ground reaction force of sufficient magnitude to be detected by the HAL shoe sensors. Initiation or discontinuation of a new gait rehabilitation program, systemic administration of steroids, and administration of riluzole, sodium valproate, or any drug intended to inhibit the progression of the target disease for which HAL was being evaluated was not permitted within two months of the pre-observation period of the trial and throughout the study period.

### Randomisation and masking

**Fig. 1:**
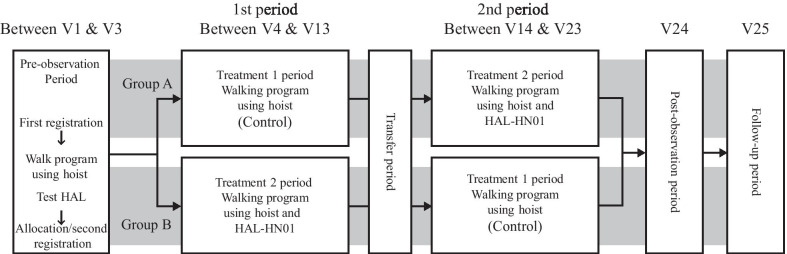
Trial schedule used to test the effectiveness of the Hybrid Assistive Limb (HAL). V, visit

The trial consisted of a four-week pre-observation period, two treatment blocks of 13 weeks each, and a four-week post-observation period. At the end of the pre-observation period, during which eligibility was assessed, the eligible patients were randomised (1:1) to either group A or B. The groups differed in the sequence of the treatments received: for group A, a control walking program with hoist only (treatment 1) was followed by a walking program with hoist and HAL (treatment 2). For group B, treatment 2 was followed by treatment 1 (Fig. 1). The patients were randomised using a web-based allocation system, with stratification for sex, age (< 65 and ≥ 65 years), and disease (SMA/distal myopathy and ‘other’ diagnoses). All the individuals were assigned numerical designations as site number-subject number (Table 1). While this was an open-label trial, individuals who performed the visual gait assessment were blinded to group allocation and patient- and disease-related characteristics.

**Table 1:** Baseline characteristics of the subjects tested in the present study

Characteristic	Group A (N = 13)	Group B (N = 11)
Age (years)	56.0 (13.2)	55.5 (7.8)
< 65	10 (76.9%)	9 (81.8%)
≥ 65	3 (23.1%)	2 (18.2%)
Female sex	6 (46.2%)	6 (54.5%)
Height (m)	1.642 (0.085)	1.643 (0.099)
Weight (kg)	54.97 (9.16)	59.15 (14.67)
Body mass index (kg/m^2^)	20.38 (2.85)	21.67 (3.89)
Blood pressure (mm Hg)	128.7 (8.5)/84.7 (13.6)	118.2 (15.6) / 72.9 (10.0)
Heart rate (beats/min)	75.0 (9.3)	75.6 (8.0)
Abnormal ECG	4 (30.8%)	5 (45.5%)
Other neurological abnormal findings	0 (0%)	0 (0%)
Muscle CT findings consistent with the target disease	13 (100%)	11 (100%)
Previous diseases	0	0
Concurrent diseases	12 (92.3%)	9 (81.8%)
Number of targeted neuromuscular diseases		
Spinal muscular atrophy	3	2
Spinal bulbar muscular atrophy	2	0
Amyotrophic lateral sclerosis	1	0
Charcot Marie Tooth disease	1	2
Myotonic dystrophy	0	2
Distal myopathy with RV	0	1
Distal myopathy, Myoshi	2	2
FSH-type muscular dystrophy	2	1
Other limb girdle-type muscular dystrophy	1	1
Sporadic inclusion body myositis	1	0
Concomitant treatment, medication	13 (100%)	11 (100%)
Other rehabilitation programs	9 (69.2%)	8 (72.7%)
Outpatient during treatment periods	8 (61.5%)	2 (18.2%)
2MWT (metres)	63.258 (30.457)	67.257 (25.508)
Barthel index (total score: 0–100)	75.4 (21.0)	84.5 (11.9)
Total Manual Muscle Test (MMT) scores (0–60)	31.12 (5.57)	30.59 (3.64)

Data are means (SD) or numbers (%); SD, standard deviation; RV and FSH denote Rimmed Vacuole and Facioscapulohumeral, respectively

### Investigational device

HAL-HN01 (Cyberdyne Inc., Tsukuba, Japan) is an investigational device distributed in the EU under the name HAL-ML05 and has received ISO13485 and CE0197 approval as the first robotic treatment medical device. HAL-HN01 was invented by one of the authors, Dr Yoshiyuki Sankai, for the recovery of gait in patients with neuromuscular diseases. HAL-HN01 is an upgrade to the previous HAL models and has an improved capacity to detect the low magnitude and sparse motor unit potentials through the skin of patients with neuromuscular disease.

Detailed device specifications for HAL-HN01 are available in the package insert and operation manual. Briefly, HAL-HN01 weighs approximately 15 kg and consists of an exoskeletal frame for the femurs, lower legs, and waist, two motor power units for each leg driven by a lithium-ion battery on the hip and knee joints in order to assist flexion and extension, a back module, a controller, and sensor shoes that measure the gait phase. There are two electrodes for each direction of joint movement and one reference electrode. In total, nine electrodes are necessary for each leg to detect the motor unit potentials on the surface of the wearer’s skin. The hybrid mechanism that enables the integration of mechanical assistance and the wearer's power for his/her own voluntary movement is incorporated within the HAL system using three control mechanisms: CVC, cybernic autonomous control (CAC), and cybernic impedance control (CIC). In CVC, the assisting torque controls movement based on bioelectric signal intensity. Bioelectric signals (BES) are motor unit potentials on the skin; they correspond to the motor torque required for each joint movement, including hip and knee, in accordance with the wearer’s motor intention. In CAC, assistance for pre-programmed leg movement patterns is provided, and these are available for each type of ideal movements (standing up to maintaining the standing position, and maintaining the standing position to walking) and serve as a guide to achieving the ideal movement pattern of the connected limbs of the wearer and HAL. In CIC, outputs corresponding to joint movements (impedance control) are provided. As a result, the wearers do not feel the additional weight of HAL or change in the moment of inertia and are able to perceive the position and movement of their legs with their own proprioception. Both CIC and CAC are always activated. The expected walking speed is input by the operator to adapt the CAC support to the gait cycle changes according to the walking speed. In the CVC, the electrodeposition, BES sensitivity, signal processing filter, and balance between the extensor and flexor assist torque are adjusted by the operator at the beginning of the gait exercise so that the wearer can walk naturally and comfortably with HAL.

All members of the medical staff, including medical doctors, physiotherapists, occupational therapists, nurses, and clinical assistants who operate the HAL-HN01 system are required to complete an education program (minimum of four hours), including a certification examination by Cyberdyne Inc. to ensure a safe use of HAL-HN01.

### Procedures and cybernic treatment

After providing informed consent, eligible patients were enrolled into the pre-observation period of the trial (Fig. 1), during which the patients wore HAL-HN01 to ensure proper functioning of the CVC and shoe sensors and were subsequently randomised to group A or B.

**Fig. 2:**
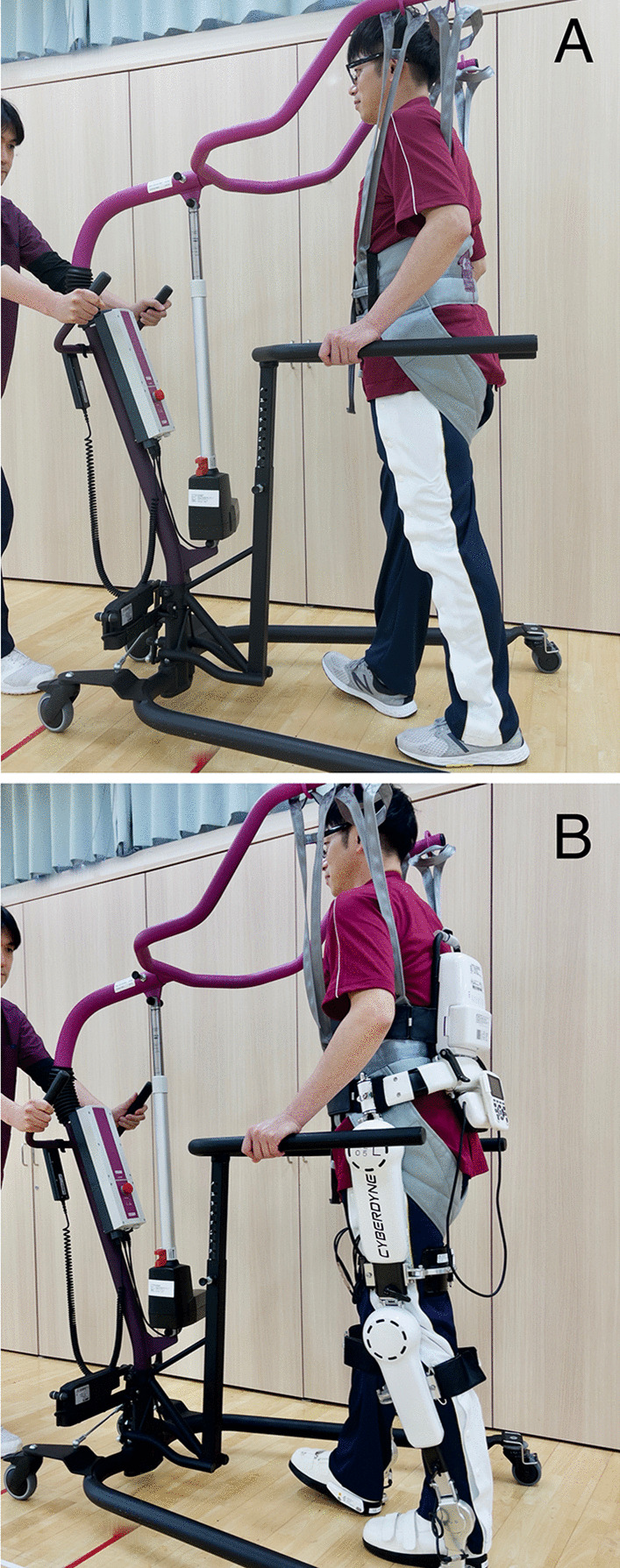
Photographs showing the use of mobile hoist and HAL-HN01 in control and cybernic treatments. **A** Control treatment (treatment 1), patient in hoist only. **B** Cybernic treatment (treatment 2), patient in hoist + wearing HAL

Treatment 1 (conventional control treatment) consisted of a 40-min walking session using only a dedicated hoist (All in One®, Ropox A/S, Denmark) (Fig. 2A). The hoist was used for safety, to prevent falls, and to facilitate the provision of any gait assistance needed by the medical staff. A single walking session comprised warming up, 20 to 30 min of walking exercise depending on the patient’s condition, a brief rest, and a period of cooling down. Treatment 2 (cybernic treatment intervention) also consisted of a 40-min walking session using a hoist, with HAL-HN01 applied to the lower limbs (Fig. 2B). For both the treatments 1 and 2, the patients in groups A and B completed the sessions in nine visits (days), up to four visits per week, over a 13-week allowance period. The alternate treatment of either treatment 2 or 1 was then completed (Fig. 1). A transfer period, from one to 3 weeks, was scheduled between the treatments 1 and 2 to prevent any interference between the treatments’ efficacies and adverse effects. At the end of this period, there was a 4-week post-observation period to observe the persistence of clinical efficacy and the occurrence of adverse events after treatment. The treatment efficacy was evaluated as the change rate in the measured outcomes as follows: from the first to the final session of treatment 1 (i.e., Visit (V) 4 and V13 for group A and V14 and V23 for group B), and from the first to the final session of treatment 2 (i.e., V14 and V23 for group A, and V4 and V13 for group B), as well as to the final session of the post-observation period (i.e., V24 for both groups).

### Outcome measures

Ambulatory function was evaluated using two standardized outcome measures: the 2-min walk test (2MWT) [37] and the 10-m walk test (10MWT) [38]. The 2MWT evaluates the maximum gait distance (metres) a patient can walk around the dedicated track circuit using the hoist for 2 min. As the 2MWT reflects both walking speed and endurance over 2 min, it was considered to be an appropriate primary endpoint to evaluate the efficacy of the cybernic treatment. The 10MWT was used to evaluate the secondary endpoints of gait speed (m/s), cadence (steps/s), and step length (m), as patients walked as fast as possible using the hoist. To evaluate the subjective effects of exercise therapy, including adverse effects, on gait improvement and to assess the impact of overwork in patients with neuromuscular diseases, a patient-reported outcome (PRO) measure of a feeling of gait was used as another secondary endpoint to evaluate five subjective perceptions: a sense of fatigue when walking, the lightness of foot when walking, a sense of stability when walking, a feeling of safety when walking, and a feeling of pleasure when walking. Each perception was quantified using a visual analogue scale (0 to 100, from worst to best) and added up for a total PRO score. The score of a sense of fatigue was subtracted from 100 to match the meaning of the other items. A retrospective pre-test that compared the previous state (V4 then and V14 then) to the present (V13 and V23, respectively), as well as pre- and post-tests (V13–V4 for the first period, V23–V14 for the second period) were performed. A visual gait analysis was performed using the video recording of the 2MWT and 10MWT to provide a quantitative evaluation of changes in stance and swing gait patterns using the modified Rivermead Visual Gait Assessment [39]. The total value was calculated by evaluating the deterioration and improvement (i.e., changes) on a 7-point Likert scale (− 3 to + 3) for 16 items of stance and swing phases. Manual muscle testing (MMT) [40], ordinal scale (0–5) of each muscle group, is a standardized set of assessments that measure muscle strength and function. Twelve MMT scores of each lower limb (bilateral flexion and extension of hip, knee, and ankle) were summed as total MMT score (0–60). The Barthel index, the standardized functional scale (0–100), was used to evaluate activities of daily living (ADL) [41]. A 12-lead electrocardiogram was used to evaluate the cardiac response to the walk exercise load. Pulse rate and blood pressure were also monitored. All adverse events and reactions were qualitatively recorded.

A three-point scale (1 = easy to use, 2 = intermediate, 3 = difficult to use) was evaluated by the operator to assess six criteria (general operability of HAL, preparation, putting on HAL, walking, taking off HAL, and maintenance). The time from attaching the electrode to the completion of putting on HAL was measured at the 5th, 7th, and 9th sessions of HAL. The safety of the device was also evaluated in terms of failure and by a review of the error history saved in the HAL system.

### Statistical analysis

The target sample size was 30 subjects (15 subjects per group). Previous clinical unpublished data indicate that the use of HAL for Well-Being (the non-medical model), which is the first HAL model without medical insurance coverage which can be used for disabled people in Japanese regulations, resulted in a clinically significant improvement (mean ± standard deviation, 28.5% ± 21.5) in gait speed and distance. Assuming no improvement in treatment 1 (hoist only), the sample size required to detect an intergroup difference (significance level, 0.05; power, 0.90) in a parallel group design would be 12 subjects per group. Typically, the required sample size for a crossover design is less than half of that of a parallel group design [42]. Nevertheless, we did not reduce the number of cases to allow for stratified allocation and to evaluate the device's safety.

The efficacy of the cybernic treatment in terms of the primary endpoint (2MWT) was evaluated by an independent evaluator according to the predefined statistical analysis plan for the trial, as per ICH E-9 guidelines. The full analysis set (FAS) used in the intention-to-treat analysis included the data from all randomised patients except those for whom a complete assessment of the primary endpoint was not performed at the end of both treatment periods (Fig. 1). The data for these patients were not included, as there was no adequate guideline for missing data in the crossover design. Data in the FAS that violated important inclusion criteria or exclusion criteria, which would have affected the results of the evaluation of the primary endpoint, were excluded from the “per protocol set” (PPS). The “all subjects as treated set” (ASaT) included data from patients who wore HAL-HN01 at least once during the study period, including the pre-observation period.

**Fig. 3:**
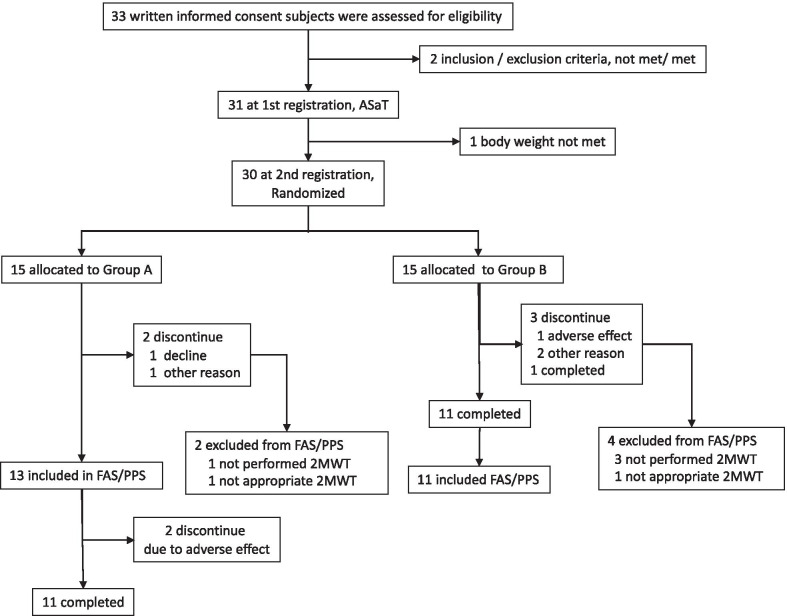
Overview of the trial for the efficacy and safety of the Hybrid Assistive Limb (HAL)

The numbers of subjects who were eligible, declined, and discontinued according to the study protocol, together with the corresponding reasons, are shown in the overview of the trial (Fig. 3).

Demographic data (age, sex, body weight, and height) and baseline values (disease diagnosis, Barthel index score, and MMT) are summarised by group in Table 1 for comparison. The mean and standard deviation were calculated for continuous variables, while ratios were calculated for the between-group comparison of categorical values.

Between-group differences were evaluated using two-tailed *t* tests for continuous variables, a Mann–Whitney *U* test for ordinal-scale variables, and Fisher’s exact test for nominal variables. A *p* value lower than 0.05 was considered significant. The treatment efficacy for the primary endpoint was evaluated using a two-sample *t* test to compare group A and group B in the crossover design [42]. First, the improvement effect (− d_A_ or d_B_) was calculated as a difference (i.e., percent change from baseline) between the values for the first and second treatment periods, where baseline was V4 for group A and V14 for group B. Hence, d_A_ or d_B_ was defined as (treatment 1 − treatmen2) in group A, and (treatment 2 − treatment 1) in group B, respectively. Reversing the sign of the values in group A was needed to indicate its HAL improvement effect. A significant treatment effect (− (d_A _− d_B_)/2) in the primary endpoint was defined as the difference between group A (d_A_) and group B (d_B_) according to the cross-over analysis method [42]. A half of the difference between groups A and B was the actual HAL effect in the cross-over comparison. It indicated the therapeutic efficacy of the cybernic treatment with HAL-HN01. We also assumed that the measured treatment effect would be comparable for each group, indicating that the effect of the intervention was independent (the carryover effect) and stable regardless of the order of the treatments 1 and 2 (the period effect). Therefore, the period effect ((d_A_ + d_B_)/2) and the carryover effect, which should not be significant, were also calculated to check this assumption [42].

Pre-planned secondary analyses, pre- and post-evaluation using one-sample *t* test for continuous variables or Wilcoxon signed-rank test for ordinal-scale variables and the intergroup evaluation of all the endpoints were carried out for the first period (V13–V4), the first and second periods (V23–V4), and for all periods, including the post-observational period (V24–V4). In those analyses, the parallel-group comparisons between treatment 1 (hoist only) and treatment 2 (HAL) were included using the first period (V13–V4) of group A and B to check for the robustness of the results in the crossover design. All statistical analyses were performed using SAS ver. 9.3 (SAS Institute; Cary, NC, USA) in Windows 7.

## Results

Of the 33 patients who provided written informed consent, two were ineligible at the time of registration to the pre-observation period (Fig. 3). The evaluation of safety was conducted in the remaining 31 patients. One patient was withdrawn from the trial during the pre-observation period, leaving 30 eligible patients at the second time point of registration. After randomisation, six patients were excluded for the following reasons: in group A, one patient voluntarily withdrew from the trial prior to the last 2MWT during the treatment period, and one patient was excluded because of an unmet 2MWT; in group B, one patient withdrew from the trial before the last 2MWT owing to pneumonia, one was excluded owing to an unmet 2MWT, and two discontinued the trial prior to the last 2MWT owing to the investigators’ judgment that it was too difficult for these subjects to continue the trial. Therefore, for the analysis of the FAS, group A consisted of 13 patients and group B consisted of 11 patients. The PPS was identical to the FAS.

The distribution of patient-related characteristics and baseline measures is summarised in Table 1. Individual patient profiles of baseline characteristics are also shown in Table 2. The groups were well balanced in the majority of relevant factors, except that group A had a higher number of patients with an outpatient status during the treatment period compared with group B (*p* < 0.047). The baseline measures for the primary endpoint of 2MWT, Barthel index, and total MMT score were also comparable between the groups (Tables 1 and 2).

**Table 2:** Individual patient profiles of baseline characteristics

Targeted neuromuscular diseases	Patient ID	Group	Age (years)	Barthel Index (0–100)	2MWT (m)	Total MMT scores (0–60)
Spinal muscular atrophy	1-3	A	52	95	116.3	35
	6-1*	A	48	100	97.3	37
	6-2*	A	57	90	66.5	30
	1-1	B	59	85	39.3	33
	9-6	B	57	60	68.6	28
Spinal bulbar muscular atrophy	1-2	A	64	80	41.9	36
	5-1*	A	54	60	30.1	39
Amyotrophic lateral sclerosis	3-2	A	69	25	30.3	26
Charcot Marie Tooth disease	9-3	A	76	95	120.1	39
	8-1	B	55	95	55.3	31
	8-2	B	63	85	126.5	37
Myotonic dystrophy	9-2	B	65	90	75.3	32
	9-4	B	51	90	90.4	28
Distal myopathy with RV	2-3*	B	40	100	87.7	24
Distal myopathy, Myoshi	2-4*	A	50	80	78.3	23
	5-3*	A	63	65	25.0	31
	2-2	B	58	90	85.2	30
	7-2*	B	47	85	59.7	36
FSH-type muscular dystrophy	1-4*	A	40	90	56.8	37
	2-7	A	46	75	57.3	29
	2-6	B	51	85	79.1	31
Other limb girdle-type muscular dystrophy	2-5*	A	33	55	97.1	26
	9-7	B	65	65	48.3	29
Sporadic inclusion body myositis	3-1*	A	76	65	61.7	34

The patient ID indicates site number-patient number. An asterisk (*) in a patient’s ID indicates the outpatient status during the study period

**Fig. 4:**
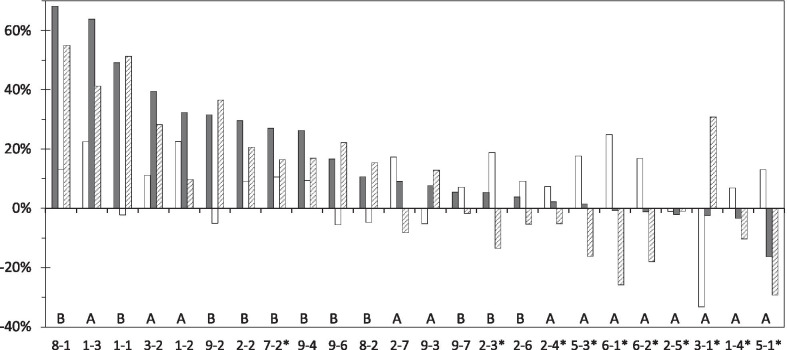
Rate of change for the two-minute walk test (2MWT) for all participants. This figure shows the rate of change (%) in the primary endpoint, the 2MWT, between the treatment 1 (control, white bars) and treatment 2 (HAL, black bars), as well as the therapeutic positive effect (stripe bar) of cybernic treatment (black bar- white bar: -d_A_ or d_B_) for group A or B. Group (A or B) and patient number are also shown. Asterisks (*) indicate the outpatient status during the study period. The order of treatments 1 and 2 was randomly determined by crossover design. Patients in the graph are displayed in order of the magnitude of the effect of treatment 2

For the FAS, the 2MWT data for 24 individuals are shown in Fig. 3. Patients in Fig. 4 are displayed in order of the magnitude of the effect of the treatment 2 (HAL). A treatment effect of HAL over the control treatment effect was observed in 13 subjects (upward stripe bar). A pre/post effect of the treatment 2 (HAL) was observed in 18 subjects (upward black bar). Six patients (6-1*, 6-2*, 2-5*, 3-1*, 1-4*, 5-1*) with the negative effect (downward black bar) of the treatment 2 (HAL) were all the outpatients in the group A. While HAL was not effective in these six patients, four of them (6-1*, 6-2*, 1-4*, 5-1*) obtained sufficient improvement with the treatment 1 (hoist only) before starting the treatment 2 (HAL). The crossover analyses using the FAS are summarised in Table 3. The treatment effect with HAL-HN01 compared with hoist only was identified for the primary endpoint (2MWT), as reflected in the mean change in the 2MWT (10.066%, 95% confidence interval: 0.667–19.464; *p* = 0.037; Table 3). The carryover and period effects were not significant (*p* = 0.331 and *p* = 0.051, respectively), thereby confirming the validity of the assumptions in the crossover design.

**Table 3:** Crossover analyses of the primary and other endpoints

Group A (N = 13) vs Group B (N = 11)	Treatment effect Mean(95% CI)	*p* value	Carryover effect Mean(95% CI)	*p* value	Period effect Mean(95% CI)	*p* value
Outcome measures						
2MWT %	10.066 (0.667, 19.464)	0.037	5.511 (− 5.982, 17.005)	0.331	− 9.371 (− 18.769, 0.027)	0.051
10MWT speed	9.140 (− 0.357, 18.636)	0.059	2.520 (− 6.919, 11.959)	0.585	− 4.623 (− 14.120, 4.873)	0.324
10MWT cadence	7.100 (2.609, 11.591)	0.003	1.373 (− 4.658, 7.405)	0.641	− 1.760 (− 6.251, 2.731)	0.425
10MWT step length	1.238 (− 4.811, 7.288)	0.675	1.369 (3.878, − 6.616)	0.594	− 2.529 (− 8.578, 3.520)	0.395
Visual gait assessment total score	1.167 (− 0.056, 2.389)	0.060	0.75 (− 0.221,1.721)	0.123	0.015 (− 1.056, 1.389)	0.780
Total MMT score*	3.04 SD (5.57)	0.039	1.81 SD (3.24)	0.148	− 2.68 SD (5.57)	0.063
Barthel index score*	0.4 SD (1.6)	0.909	0.0 SD (2.4)	0.675	− 0.9 SD (1.6)	0.119
PRO total score (Post–pre)	− 5.294 (− 70.379, 59.792)	0.868	− 7.3217 (− 84.858, 70.2150)	0.847	− 23.4755 (− 88.561, 41.610)	0.462
PRO total score (Post−then)	4.032 (− 52.215, 60.278)	0.883	23.101 (− 68.065, 114.267)	0.604	− 31.877 (− 88.124, 24.369)	0.252
Response shift total score (Pre−then)	9.325 (− 42.263, 60.913)	0.711	30.423 (− 36.371, 97.217)	0.355	− 8.402 (− 59.990, 43.186)	0.739

*p* value was calculated using a two-sample t test or Mann–Whitney U test*; CI, confidence interval; SD, standard deviation; 2MWT, two-minute walk test; 10MWT, ten-metre walk test; MMT, manual muscle test; PRO, patient-reported outcome

Regarding other endpoints, the percent changes in cadence at 10MWT and in total MMT scores indicated significant improvement (*p* = 0.003 and *p* = 0.039, respectively) as a result of cybernic treatment with HAL in the crossover design. Differences in speed at 10MWT were substantial but not statistically significant (*p* = 0.059), while step length was not significantly different. Barthel index score, PRO total score (post–pre), PRO total score (post-then), and response shift total score (pre-then) exhibited no significant improvement in the treatment 2 (HAL) compared with the treatment 1 (hoist only) in the crossover design.

In the parallel group comparisons (V13–V4) between groups A and B in the first period (Table 4), the distance for 2MWT (15.577%, *p* = 0.044) and cadence (8.474%, *p* = 0.026) were significantly higher in treatment 2 (HAL) compared with treatment 1 (hoist only). Moreover, in the post-/pre-evaluations in the first period, 2MWT distance, gait speed, cadence, step length, and total MMT score were significantly higher only in treatment 2.

**Table 4:** Changes in primary and secondary endpoints over the course of the study periods

	First treatment period (V13–V4)						First + second treatment periods (V23–V4)					
	Treatment 1 in Group A (Control) N = 13	*p* value*	Treatment 2 in Group B (HAL) N = 11	*p* value*	Treatment 2 vs. Treatment 1 N = 11,13	*p* value**	Group A (Control + HAL) N = 13	*p* value*	Group B (HAL + Control) N = 11	*p* value*	Group A vs. Group B N = 13,11	*p* value**
	Mean (95% CI)		Mean (95% CI)		Mean (95% CI)		Mean (95% CI)		Mean (95% CI)		Mean (95% CI)	
2MWT Distance	9.297 (− 0.153, 18.746)	0.053	24.874 (11.439, 38.309)	0.021	15.577 (0.494, 30.660)	0.044	16.808 (4.694, 28.922)	0.011	41.944 (17.266, 66.622)	0.004	− 25.14 (− 49.650, − 0.621)	0.045
10MWT Speed	7.706 (− 1.327, 16.740)	0.088	19.366 (8.870, 29.863)	0.002	11.660 (− 1.304, 24.624)	0.075	17.321 (4.776, 29.867)	0.011	30.951 (18.934, 42.969)	0.0002	− 13.630 (− 30.204, 2.943)	0.102
10MWT Cadence	2.802 (− 2.928, 8.533)	0.308	11.276 (6.221, 16.331)	0.0006	8.474 (1.127, 15.820)	0.026	10.603 (0.461, 20.746)	0.042	13.270 (7.619, 18.921)	0.0004	− 2.661(− 14.249, 8.914)	0.638
10MWT Step length	4.555 (− 0.717, 9.828)	0.084	7.163 (0.228, 14.098)	0.044	2.607 (− 5.451, 10.666)	0.509	6.176 (0.335, 12.016)	0.040	15.581 (7.102, 24.059)	0.002	− 9.405(− 18.84, 0.031)	0.051
Visual gait assessment total score	− 0.462 SD(3.126)	n/a	1.000 SD(1.483)	n/a	1.462 (− 0.676, 3.599)	0.17						
MMT total score	0.23 (− 1.46, 1.92)	0.844^†^	1.77 (0.34, 3.20)	0.016^†^	1.54(− 0.60,3.68)	0.150^††^	0.77 (− 0.31, 1.85)	0.191^†^	1.91 (0.40, 3.42)	0.023^†^	− 1.14 (− 2.85, 0.57)	0.180^††^
Barthel index score	1.2 (− 0.2, 2.5)	0.250^†^	1.4 (− 0.8, 3.5)	0.500^†^	0.2 (− 2.1,2.5)	0.902^††^	1.5 (− 0.4, 3.4)	0.250^†^	1.4 (− 0.8, 3.5)	0.500^†^	0.2 (− 2.5, 2.9)	0.838^††^
PRO total score (Post–Pre)	101.6 (4.2, 199.0)	0.042	89.0 (19.5, 158.5)	0.017	− 12.6 (− 129.9, 104.6)	0.825	128.5 (30.6, 226.5)	0.014	121.9 (46.9, 196.9)	0.005	6.6 (− 113.5, 126.8)	0.9099
PRO total score (Post-Then)	80.2 (3.1, 157.4)	0.042	107.4 (30.2, 184.5)	0.011	27.1 (− 76.5, 130.8)	0.593						

	All periods (V24–V4)					
	Group A (Control + HAL) N = 12	*p* value*	Group B (HAL + Control) N = 11	*p* value*	Group A vs. Group B N = 12,11	*p* value**
	Mean (95% CI)		Mean (95% CI)		Mean (95% CI)	
2MWT Distance	12.534 (− 3.505, 28.573)	0.113	35.594 (11.276, 59.911)	0.0086	− 23.059 (− 49.924, 3.805)	0.089
10MWT Speed	18.180 (− 0.853, 37.212)	0.060	27.429 (13.160, 41.699)	0.0016	− 9.250 (− 31.967, 13.467)	0.407
10MWT Cadence	9.190 (− 2.3335, 20.714)	0.107	11.04 (6.9550, 15.125)	0.0001	− 1.850 (− 13.813, 10.113)	0.751
10MWT Step length	7.486 (− 0.235, 15.206)	0.056	14.680 (3.060, 26.301)	0.0183	− 7.195 (− 20.065, 5.675)	0.258
Visual gait assessment total score						
MMT total score	0.46 (− 1.29, 2.22)	0.605^†^	2.05 (0.36, 3.73)	0.027^†^	− 1.58 (− 3.91, 0.74)	0.171^††^
Barthel index score	1.5 (− 0.4, 3.4)	0.250^†^	0.9 (− 0.4, 2.3)	0.500^†^	0.6 (− 1.7, 2.9)	0.743^††^
PRO total score (Post–pre)						
PRO total score (Post–then)						

^*^p value was calculated using a one-sample *t* test or ^†^Wilcoxon signed-rank test

^**^p value was calculated using a two-sample *t* test or ^††^Mann–Whitney *U* test

SD, standard deviation; CI, Confidence Interval; 2MWT, two-minute walk test; 10MWT, ten-metre walk test; MMT, manual muscle test; PRO, patient-reported outcome

In V23–V4 (first + second treatment periods, Fig. 1, Table 4), the 2MWT distance, speed, cadence, and step length exhibited significant changes in comparison to the post-/pre-evaluation of both groups A and B. In this comparison, total MMT score showed significant changes only in group B. In V23–V4 (first + second treatment periods), the 2MWT distance for group A was significantly lower than that of group B (Table 4). The aforementioned trends lasted until the post-observational period in a manner comparable to that of all periods.

The PRO total score exhibited significant changes in comparison to post/pre and post/then in the first period and the first + second periods (Table 4). However, there was no significant difference in PRO between treatments 1 and 2 in the first period. The Barthel index showed no significant changes in comparing the post/pre evaluation and between groups in the first, first + second, or all periods.

For the 5th use of HAL-HN01 and later, the three point evaluation score improved significantly in comparison to both first time use in overall and all six criteria (Table 5). The response "difficult to use" was not found after the 7th use of HAL-HN01. The time taken to put on HAL at the 5th use had an average value of 323.5 s, a median of 233.5 s, a minimum of 110 s, and a maximum 1200 s, and exhibited a wide degree of variation between clinical sites. While the time taken to put on HAL tended to decrease each time, the differences were not significant.

**Table 5:** Changes in the time (s) taken to put on HAL from the 5th time of use of HAL-HN01

Times of use of HAL-HN01	5th		7th		9th		7th–5th		9th–5th	
	Mean median	(SD) (min, max)	mean median	(SD) (min, max)	mean median	(SD) (min, max)	Mean (95% CI)	*p* value	mean (95% CI)	*p* value
Time to wear HAL (seconds)	323.5 233.5	(245.3) (110, 1200)	293.3 244.5	(221.5) (125, 1080)	281.0 235.5	(199.7) (123, 1080)	− 30.2 (− 87.3, 26.9)	0.2853	− 42.5 (− 99.2, 14.2)	0.1346

SD, standard deviation; CI, Confidence Interval; min, minimum; max, maximum

Safety analyses were performed for all 31 subjects included in the ASaT analysis (Fig. 3). In one subject who wore HAL once only during the pre-observation period, no adverse event or device failure occurred. In the 30 randomised patients (Fig. 3), adverse events occurred in 55.2% (16/29 subjects, 31 events) in treatment 1 (hoist only) and in 80.0% (24/30 subjects, 42 events) in treatment 2 (HAL). There was no significant difference (*p* = 0.0539, Fisher’s exact test) in total adverse event's frequency between treatments 1 and 2. The incidence rate of adverse events associated with the use of HAL was 46.7% (14/30 patients), and a total of 19 HAL-related adverse events were recorded, mostly in treatment 2 (Table 6). All the adverse events were mild and were easily remedied. Of the adverse events, pain (myalgia, back pain, arthralgia, pain in extremity, as well as arthrosis deformans pain) associated with walking programs accounted for approximately a half of all recorded adverse events. Contact dermatitis, excoriation, erythema, and skin exfoliation were common owing to contact with electrodes and cuffs of HAL. Falls and contusions occurred when HAL was not in use, but they were reported by an investigator to be related to the HAL-HN01 because they happened during the treatment 2 period. In addition, there were no adverse events associated with a failure of the device.

**Table 6:** Incidence of causally-related adverse events during the trial period of the present study

Adverse event	Main cause	Incidence %	Events
Overall patient number = 14 of 30		46.7% (total patients)	19 (total events)
Myalgia	Walk program, weight of the device itself	13.3%	4
Contact dermatitis	Contact with the electrodes	10.0%	3
Back pain	Walk program, contact with the back module	6.7%	2
Excoriation	Contact with the cuffs	6.7%	2
Erythema	Contact with the electrodes	3.3%	1
Skin exfoliation	Contact with the electrodes	3.3%	1
Arthralgia	Walk program	3.3%	1
Arthrosis deformans pain	Walk program	3.3%	1
Pain in extremity	Walk program	3.3%	1
Pain	Walk program	3.3%	1
Fall	No occurrence in use but causality cannot be denied	3.3%	1
Contusion	No occurrence in use but causality cannot be denied	3.3%	1

The summary technical document containing the aforementioned data has been successfully reviewed by the PMDA. HAL-HN01 has been approved as a new medical device (approved number: 22700BZX00366000, November 25, 2015) by the Ministry of Health, Labour, and Welfare and is covered by general health insurance in Japan for the treatment of patients with neuromuscular diseases.

## Discussion

The present RCT sought to evaluate the cybernics treatment's efficacy and safety to improve the ambulatory function in eight rare neuromuscular diseases. The crossover analysis confirmed a significant effect of the cybernic treatment on the primary endpoint measure (2MWT), which showed a 10.066% increase compared with the control treatment (hoist only). The results of this trial confirmed that patients with ambulatory dysfunction owing to neuromuscular diseases can achieve significant improvement in the gait function by performing at least nine 40-min sessions of intermittent cybernic treatment with HAL-HN01.

The eligible participants were enrolled into the pre-observation period of the trial and randomised into one of the two groups, so FAS was determined (Fig. 3). In the case of the crossover design (Fig. 1), there are no statistical methods to properly complement the missing values. Therefore, it was appropriate to exclude subjects with partial completion of the 2MWT by the pre-defined criteria of FAS (Fig. 3). Groups A and B were balanced regarding all the baseline measures except for the outpatient status during treatment (Table 1). Eight subjects from group A underwent the trial as outpatients. As six of eight subjects exhibited a higher therapeutic effect of the control treatment than the HAL treatment (Fig. 4), we determined that the effect of treatment with HAL was not overestimated. There were no influences of baseline characteristics on the crossover analyses of treatment effects for groups A and B (Table 1).

Our study showed that the 2MWT was an appropriate indicator of ambulatory capacity in patients with neuromuscular diseases. While previous studies often used the six-minute walk test (6MWT) to assess the submaximal capacity for functional walking that is necessary for ADL across different disease states [37], the 2MWT can reduce high inter-test variability related to fluctuating motivation observed with the 6MWT and is a valid alternative to the 6MWT [37]. The 2MWT has been able to eliminate the problem of extreme muscle fatigue following 6MWT for subjects with neuromuscular diseases [5, 6].

As neuromuscular diseases are both progressive and incurable, and as aetiologically-based drugs for neuromuscular diseases have not been shown to stimulate neuromuscular reconnection or remodulation, our results demonstrating the successful intervention of cybernic treatment are truly ground-breaking [36]. The data for secondary endpoints in the present study demonstrated that cybernic treatment significantly improved cadence. Since we did not observe a significant change in walking speed, cybernic treatment may have alternatively influenced walking endurance as indicated by 2MWT. In addition, we also consider the significant improvement in the total MMT score after cybernic treatment to be groundbreaking. The present results dispel the notion that the use of HAL for neuromuscular diseases might be also an excessive and dangerous exercise therapy [5, 6]. Thus, to the best of our knowledge, HAL is the very first intervention to improve MMT in cases of neuromuscular disease [43]. Furthermore, we also observed substantial improvements in walking patterns in a visual gait analysis although differences were not significant. As ADL measured by the Barthel index did not significantly improve after nine cybernic treatments in this trial, further trials should investigate whether ADL can be improved by increasing the number of cybernic treatments. In addition, the PRO total scores did not significantly improve during our trial. As PRO scores were subjectively evaluated, recording these values at each of the four-time points, i.e., pre and post in the first and second periods in the crossover design, may have exceeded the limits of patients’ subjective measurement capacity. In the first period (V13–V4), parallel comparisons of groups A and B between the control treatment (treatment 1) and HAL (treatment 2) reaffirmed the primary crossover analyses’ robustness. Both the treatment 1 (hoist only) and treatment 2 (HAL) were performed for group A or B in the opposite order in V23–V4. The results showed that starting with the treatment 2 (HAL) first, followed by the control treatment (group B), led to a more significant improvement in 2MWT distance. These data may suggest that if the same number and content of cybernic treatment (HAL) sessions are performed, the earlier the cybernic treatment is given (such as in group B), the better results might be obtained. The PRO improvement effect of both the treatment 1 (hoist only) and 2 (HAL) on post–pre and post-then in the first period (V13–V4), and on post–pre in the first + second period (V24–V4) in group A and B was clearly significant. Excessive exercise therapy for patients with neuromuscular disease has been considered risky in the presence of overwork muscle weakness[5, 6]. Still, both treatment 1 and 2 improved patients' subjective ratings of the total PRO scores, indicating more comfort while walking, with no deterioration thought to be due to overwork. This may be because patients with neuromuscular diseases but a willingness to walk evaluate the walking exercise with hoist as safe and good, regardless of whether they use HAL or not. It might also indicate the absence of gait exercise in the present standard clinical care for patients with neuromuscular diseases.

The time needed to put on HAL was approximately 5 min on average, indicating that suiting up with HAL does not pose a substantial burden for patients and operators for clinical use. While the time necessary to put on HAL did not decrease over time, the ease of use steadily improved according to operator evaluations. The 5th time that HAL was worn, the time to put HAL on varied widely from a minimum of 110 s to a maximum of 1200 s, and no further improvements were noted by the 9th time, indicating that sufficient education/training before using HAL is necessary rather than natural learning.

In the present study, cybernic treatment with HAL did not cause serious adverse events or secondary muscle weakness owing to the excessive use of muscles in these patients. Myalgia and arthralgia, which were observed after cybernic treatment in the present study, are common after exercise and are manageable. As a general precaution when using HAL, it is necessary to observe and address the skin conditions that result from contact with electrodes and cuffs. In particular, it is necessary to pay attention to the effects of HAL on the skin in subjects with sensory impairment, as HAL electrodes are common products used for measuring bioelectric signals, such as in electrocardiogram, and there is little room for improvement in the electrodes themselves.

In this trial, we showed that nine cybernic treatments were safe and effective for patients with neuromuscular disease. Since iBF is a method of motor learning, it is generally considered that Hebb's law can be applied. However, it should be further explored at what point the learning effect of repeated cybernic treatments reaches a plateau. More specifically, it will be necessary to clarify how the number and frequency of treatments affect this learning curve. Observational studies to this end have already begun [44].

In order to elucidate the principle of effects of cybernic treatment, the underlying mechanisms of iBF should be examined for each component involved in locomotor function. The important components that control locomotor function include the skeletal system, motor unit, proprioceptive system, spinal locomotor central pattern generator [45], and other components of the central nervous system. The reactivation of the spinal locomotor central pattern generator will need to be investigated, as a mere repetitive walking exercise may not activate this centre.

It will be necessary to show proper motor innervation of muscle fibres when examining motor units following cybernic treatment. Stable walking under a variety of conditions requires the CNS to function based on voluntary motor intention [31, 32]. Therefore, it will be necessary to examine the reconnectivity between the CNS and motor units (i.e. neuroplasticity) [34] and to noninvasively elucidate the mechanisms responsible for improvements observed with the cybernic treatment [46]. We believe that the combined cybernic treatment with nusinersen therapy in SMA, leuprorelin administration in SBMA, and exon skip therapy in DMD would show synergistic effects and further improve the treatment effects compared with each monotherapy.

Even if aetiologically-based treatments, such as antisense nucleotide therapy, are performed several years after the onset of neuromuscular disease, the motor function has been shown not to improve despite improved biomarker levels and no further worsening of symptoms [12, 13]. In this case, aetiologically-based therapies combined with cybernic treatment may have significant pre/post effects.

In the targeted population in this trial, each disease entity is rare, and these patients are vulnerable. We combined eight neuromuscular diseases into a group as neuromuscular diseases that share the same motor unit involvement. This grouping allowed us to perform a randomised controlled study of HAL-HN01 for such patients. The limitations were a short-term trial, the small number of patients with each of the diseases, and the inability to stratify the allocation of patients across all the conditions. Thus, in the future, we would like to conduct an observational study of long-term efficacy and safety in a higher number of patients with each of the diseases.

## Conclusions

The present study is the first RCT to show the effectiveness and safety of cybernic treatment for neuromuscular disease, and it provides hope for the effective treatment in those living with often debilitating and incurable neuromuscular disorders. This study showed significant improvements in gait function in patients with incurable neuromuscular diseases after nine iBF-based cybernic treatments with HAL-HN01 compared with walking exercises with a hoist only. We believe that the observed clinical effects, which are limited to improvement in gait function and total MMT score, stem from motor learning or functional regeneration by iBF. Cybernic treatment with HAL-HN01 has the potential to become standard treatment for walking impairment in patients with neuromuscular diseases, both with and without aetiologically-based therapies.

Lastly, the efficacy of iBF-based cybernic treatment will be undoubtedly verified by integrating the results of our next trial on spastic paraplegia caused by CNS lesions (NCY-2001) with those of the present study (NCY-3001). We consider that cybernic treatment can be used for a broad range of lesions and aetiologies, including those in the brain, spinal cord, and motor units, from acute to chronic stages [47]. Preliminary studies have also shown that HAL-HN01 is a useful medical device for improving the motor function. Future RCTs will be needed to prove efficacy and safety in other diseases, such as cerebrovascular disease [48–50], spinal cord injury [51, 52], multiple sclerosis, Parkinson disease, spinocerebellar degeneration, and Guillain–Barre syndrome. Although the present RCT results are limited to only neuromuscular diseases, this study opens the possibility of applying cybernic treatment to other diseases. Our findings set a precedent for replacing existing walking exercise therapy using a hoist or treadmill alone with cybernic treatment using HAL.

## Data Availability

Data sharing is not applicable to this article as no datasets were generated during the current study. Please contact the corresponding author for data requests.
